# Low prevalence of dihydro folate reductase (*dhfr*) and dihydropteroate synthase (*dhps*) quadruple and quintuple mutant alleles associated with SP resistance in *Plasmodium vivax* isolates of West Bengal, India

**DOI:** 10.1186/s12936-016-1445-9

**Published:** 2016-08-02

**Authors:** Sabyasachi Das, Abhijit Banik, Amiya Kumar Hati, Somenath Roy

**Affiliations:** 1 Immunology and Microbiology Laboratory, Department of Human Physiology with Community Health, Vidyasagar University, Midnapore, 721 102 West Bengal India; 2 Division of Parasitology, Department of Medical Entomology and Chairman, Calcutta School of Tropical Medicine, Chittaranjan Avenue, Kolkata, 700 073 West Bengal India

**Keywords:** *Plasmodium vivax*, Sulfadoxine–pyrimethamine resistance, *pvdhps*, *pvdhfr*, Single nucleotide polymorphism

## Abstract

**Background:**

Emergence of chloroquine resistant *Plasmodium vivax* is a serious obstacle towards malaria control in India. This study elucidates the temporal pattern of antifolate [sulfadoxine–pyrimethamine (SP)] resistance in *P. vivax* infection by means of genetic polymorphisms, especially analysing the single nucleotide polymorphisms of dihydrofolate reductase (*pvdhfr)* and dihydropteroate synthase (*pvdhps)* gene among the field isolates of urban Kolkata Municipal Corporation and rural Purulia region of West Bengal, India.

**Methods:**

Blood samples were collected from 99 microscopically diagnosed *P. vivax* patients (52 from Kolkata Municipal Corporation and 47 from Purulia). Parasitic DNA was extracted followed by polymerase chain reaction and sequencing of different codons of *pvdhfr* gene (15, 33, 50, 57, 58, 61, 64, 117, and 173 codons) and *pvdhps* gene (373, 380, 382, 383, 384, 512, 553, 585, and 601 codons) were performed to identify the mutations.

**Results:**

*Prevalence of* double mutant *dhfr* A_15_P_33_N_50_F_57_**R**_**58**_T_61_V_64_**N**_**117**_I_173_ allele (53.85 %) was observed in Kolkata Municipal Corporation (KMC) whereas in Purulia, wild *dhfr* A_15_P_33_N_50_F_57_S_58_T_61_V_64_S_117_I_173_ allele was predominated (48.94 %). In *pvdhps* gene a significant number of isolates (17.31 %) in KMC contained the double mutant S_373_E_380_S_382_**G**_**383**_P_384_K_512_**G**_**553**_V_585_M_601_ allele. *pvdhfr* and *pvdhps* combination haplotype revealed the emergence of quadruple (13.46 %) and quintuple (3.84 %) mutant allele in KMC, which might result in poor clinical response against antifolate drugs.

**Conclusion:**

The study reveals that *P. vivax* parasites in rural Purulia may still be susceptible to SP but additional caution should be taken for treatment of vivax malaria in KMC to limit the blooming of quadruple and quintuple mutant allele in the remainder of the West Bengal, India.

## Background

Malaria has been the major public health problem for the past few decades in West Bengal, Eastern India where both *Plasmodium falciparum* and *Plasmodium vivax* are equally prevalent. In 2013, 0.88 million people were affected by malaria, and of them 0.46 million patients were *P. falciparum* positive and 0.42 million patients were affected by *P. vivax* infection [1]. Although *P. falciparum* is the most deadly infection resulting in malignant malaria globally, *P. vivax* is the most widespread species in Southeast Asia, including India, causing severity and morbidity [2–5]. In 2009, artesunate plus sulfadoxine–pyrimethamine (SP) combination was recommended as the first-line drug against uncomplicated falciparum malaria, while chloroquine along with primaquine remains the first-line drug against vivax malaria in India [6]. The persistence of vast numbers of mixed *P. falciparum* and *P. vivax* infection in India is one of the major disease burdens as it is not easily discriminated by microscopy or rapid diagnostic test kits (RDTs). Consequently a large proportion of *P. vivax* parasites are often involuntarily exposed to SP drug pressure, resulting in the evolution of SP-resistant *P. vivax* parasites [7]. The target of pyrimethamine and sulfadoxine are respectively dihydrofolate reductase (DHFR) and dihydropteroate synthase (DHPS), two major proteins of the folate biosynthesis pathway of parasites [8, 9]. Polymorphisms surrounded by the genes that encode these active enzymes are the major factor in SP resistance. In the case of *P. falciparum* infection, SP resistance has been predominantly observed with the polymorphism of dihydrofolate reductase (*dhfr*) and dihydropteroate synthase (*dhps*) genes, throughout the globe [8–13]. Similarly, polymorphisms in *pvdhfr* and *pvdhps* genes are proven to be linked with antifolate resistance in *P. vivax* infection. Polymorphism in *pvdhfr* S58R and S117N are highly associated with pyrimethamine resistance; additional mutation in P33L, N50I, F57L, T61M, V64L, and I173L codons increases the degree of resistance, i.e., very high IC50 values for pyrimethamine [14–17]. Different field studies suggest that polymorphism at A383G and A553G of *pvdhps* gene are solely responsible for sulfadoxine resistance while additional mutations at S373T, E380K, S382A, P384L, K512E, V585G, and M601I codons confer higher levels of resistance [18, 19]. Predominance of *pvdhfr* codon S58R and S117N polymorphisms and *pvdhps* codon A383G mutation in clinical isolates was reported in studies, mainly from central, western, northern and south eastern regions of India [7, 20, 21], before the introduction of a new national drug policy. Very few studies are initiated in this part of eastern India although West Bengal is a malaria-endemic zone [7]. In 2010, 1.6 million confirmed malaria cases were reported in India; 134,795 patients were from West Bengal [1, 22].

In such settings, molecular markers involved in SP resistance in *P. vivax* infection need to be evaluated after 5 years of a new national drug policy, help to understand the current scenario of antifolate resistance in *P. vivax*, as both *Plasmodium* spp. are predominate in this part of India, resulting in a rapid admixture of parasite population with selection pressure of drug. Prevalence of double or triple *pfdhfr* (A**I**_**51**_C**N**_**108**_I or A**I**_**51**_**R**_**59**_**N**_**108**_I) and triple or quadruple *pfdhps* (**A**_**436**_**G**_**437**_**E**_**540**_AA or **A**_**436**_**G**_**437**_**E**_**540**_A**T**_**613**_) mutation was observed in early 2012 and 2013, in the same study sites of West Bengal, India, which surpassed the antifolate resistant (both in vitro and in vivo) *P. falciparum* disease burden in West Bengal, India. This alarming sign for malaria control might be due to population migration and probable admixture of different ethnic groups [10–12].

The present investigation aimed to assess polymorphisms of *pvdhfr* and *pvdhps* genes to identify the temporal pattern of antifolate resistance among field isolates of urban Kolkata Municipal Corporation (KMC) and rural Purulia. This study might also identify combinations of different mutations that may lead to different qualitative and quantitative multidrug-resistant phenotypes. This knowledge of haplotype variance of candidate gene is of importance for the adoption of future chemotherapy to surmount drug-resistant malaria.

## Methods

### Chemical and reagents

Phenol: chloroform: isoamyl alcohol, chloroform: isoamyl alcohols, agarose, p-amino benzoic acid-free RPMI 1640 were purchased from Himedia, India. Tris–Hcl, Tris buffer, potassium dihydrogen phosphate (KH_2_PO_4_), dipotassium hydrogen phosphate (K_2_HPO_4_), ethylene diamine tetra acetate (EDTA), sodium bicarbonate (NaHCO_3_), sodium acetate, ammonium acetate, ethanol, boric acid, glacial acetic acid were procured from Merck Ltd, SRL Pvt, Ltd, Mumbai, India. Proteinases K, RNase A, ethidium bromide were purchased from Sigma Chemical Co, USA. Chloroquine tablets were procured from Resochin: Bayer. Oligonucleotide primers were purchased from New England Biolabs, USA. PCR grade nucleotide mixture, MgCl_2_, dNTPs and Taq DNA polymerase were purchased from Roche, USA. *lambda exonuclease* was purchased from Gibco-BRL Life Technologies, France. pLDH kit was purchased from Diatek, Kolkata, India. All other chemicals were purchased from Merck Ltd, Mumbai and were of the highest grade available.

### Study area

**Fig. 1:**
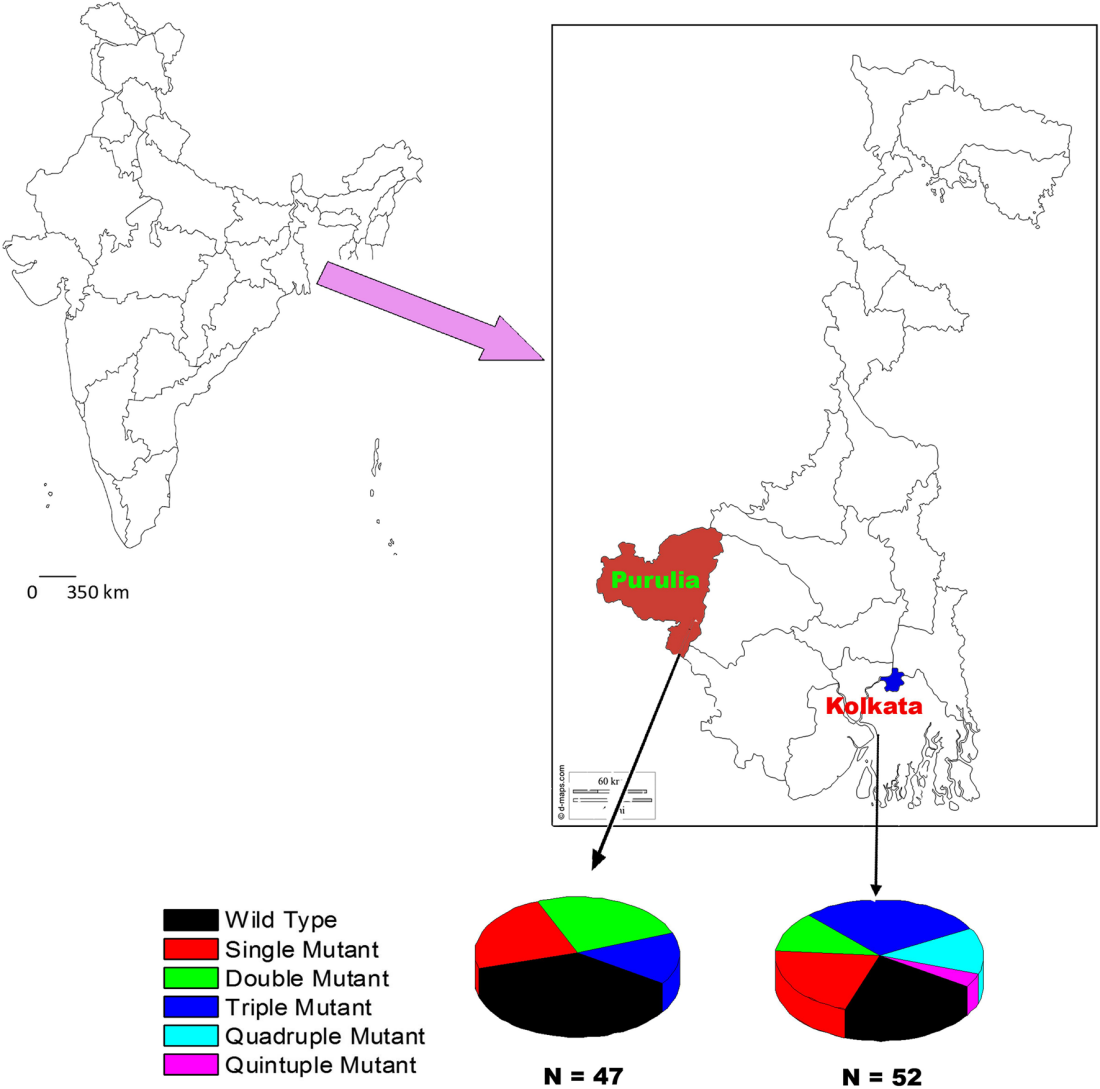
Graphical presentation of different study site with different combination genotype

This study was conducted from December 2013 to November 2014 in KMC and Purulia, two malaria-endemic regions of West Bengal, India (Fig. 1). Kolkata is the main commercial and financial hub of east and northeast India, mainly comprised of service industries, business community members, industrial and manufacturing members of a high socio-economic status, whereas Purulia is a hilltop, rural, forest area, where the majority of the population are farmers and labourers with low socio-economic status. In 2010, KMC contributed the highest malaria incidences (96,693) as well as the highest slide-positive rate (SPR) (27.21 %), the majority (82,467) being *P. vivax* infection [1, 22]. The highest number of mixed *P. falciparum* + *P. vivax* co-infection (543) was reported from KMC region, whereas in Purulia, another malaria-endemic region, *P. falciparum* was prevalent (70.13 % of 4526 malaria-positive cases, 3174 cases of *P. falciparum* infection), with no *P falciparum* + *P. vivax* co-infection [22]. Details of epidemiological information on the study sites are reported elsewhere [22].

### Selection of subjects

The criteria for selection to conduct the experiments included history of fever during previous 24 h; mono-infection with *P. vivax* based on microscopic examination of Giemsa-stained thin and thick blood smears; an RDT based on detection of *Plasmodium*-specific lactate dehydrogenase (pLDH) (OptiMAL-DT) having parasite density of 1000–200,000 asexual parasites/µl blood; and no recent history of self-medication with anti-malarial drugs. 18S rRNA gene, *pvcsp* gene was amplified by nested PCR to identify *P. vivax* infection and rule out other mixed species infection [23, 24]. Patients with signs and symptoms of severe, complicated malaria, pregnant and lactating women, infants (under 2 years old), and those with haematocrit <20 %, were excluded [25]. Informed consent was taken from all the patients and consent of a guardian was taken for children. Experimental design and protocol of this study was duly approved by the Vidyasagar University ethical committee.

### Sampling design

The sample size for the experiment was determined as described by Pal and colleagues [26], and by the standard formula (n = z^2^pq d^−2^). The minimum estimated sample size for each year was 31 [(1.96^2^ × 0.00720 × 0.9928)/0.03^2^)]. The calculation was based on the incidence of annual parasitological index (API) of malaria in India. In 2013 API was recorded as 0.72 by the National Vector Borne Disease Control Programme (NVBDCP) [1]. The desired precision (d) was 3, where, API value was serve as p, q = p − 1 and z = 1.96. A total of 52 patients from KMC and 47 patients from Purulia (age ranging from 3 to 76 years) were included in this study. *Plasmodium vivax*-positive patients received standard 10 mg/kg chloroquine (CQ) on days 1 and 2, 5 mg/kg CQ on day 3, and 0.25 mg/kg primaquine daily for 14 days (primaquine is contra-indicated in G6PD-deficient patients) under the supervision of Prof Amiya Kumar Hati (ex-Director School of Tropical Medicine, Kolkata) and his team, as recommended by NVBDCP [6].

### Separation of red blood cells

Two ml of venous blood was collected from each of patient in a vacutainer (BD Falcon) coated with an anticoagulant (EDTA). Red blood cells (RBCs) were separated using Histopaque 1077 density gradient followed by centrifugation at 1450 rpm for 45 min at 4 °C. An aliquot of approximately 1 ml of RBC pellet was obtained. Finally, erythrocytes were washed three times in folate and *p*-amino benzoic acid-free RPMI 1640 medium and stored at −20 °C for further analysis [27].

### Parasite DNA isolation

Parasite DNA was extracted from 1 ml (approximately) of infected RBC using the phenol–chloroform extraction method as described elsewhere [27]. After air drying, the extracted parasite DNA was re-suspended in TE buffer (10 mM Tris, 1 mM EDTA, pH 8.4) and stored at −20 °C until further use. Isolated DNA was quantified by 1.2 % agarose gel electrophoresis. The purity of the parasite DNA was checked spectrophotometrically by calculating the A260/A280 ratio where A280 values determine protein impurities.

### Primer designing and PCR amplification of *pvdhfr* and *pvdhps* genes

Point mutations in different variants of *pvdhfr* and *pvdhps* genes were investigated in all *P. vivax* isolates by nested PCR reactions, followed by sequencing analysis. Primers were designed on the basis of complete *P. vivax* strain sequence (accession number X98123 for *pfdhfr* and AY186730 for *pvdhps*) available in the GenBank. Approximately 200 ng of genomic parasite DNA, 10 pmol of primers, 1X reaction buffer (10 mM Tris, 50 mM KCl, pH 8.4), 2.7 mM MgCl_2_, 150 μM dNTPs, and 1 unit of Taq DNA polymerase were used to prepare 25 μl reaction mixture (master mix). PCR cycle conditions were varied in different genes. Detailed primer sequence and cycle conditions are shown in Table 1. In nest-I reaction, *pvdhfr* gene was amplified by A1F and A1R primer pair whereas 2A F and 2A R primer was used to amplify the *pvdhps* gene. The amplicon produced by NEST I reaction was used as the template DNA in NEST II reaction. All PCR amplifications contained a positive control (genomic DNA from quality control 3D7 strain) and a negative control (no target DNA).

**Table 1:** PCR primer sequences and reaction conditions were used for the amplification of sequences encoding *Plasmodium vivax dhfr* and *dhps* gene

Gene	Primer name	Primer sequence	Product size (bp)	PCR cycling conditions
Nest I				
*pvdhfr*	A1F	5′ATGGAGGACCTTTCAGATGTATTTGACATT 3′	720	105 °C for 5 min; (95 °C for 30 s; 50 °C for 30 s; 72 °C for 50 s) × 30 cycles; 72 °C for 10 min
	A1R	5′GGCGGCCATCTCCATGGTTATTTTATCGTG 3′		
*pvdhps*	2A F	5′ATTCCAGAGTATAAGCACAGCACATTTGAG3′	840	104 °C for 5 min; (95 °C for 50 s; 57 °C for 1 min; 72 °C for 1 min) × 35 cycles; 72 °C for 10 min
	2A R	5′CTAAGGTTGATGTATCCTTGTGAGCACATC 3′		
Nest II				
*pvdhfr*	A2F	5′TTTATGATGGAACAAGACTGGGACGTT 3′	701	100 °C for 5 min; (95 °C for 45 s; 52 °C for 40 s; 72 °C for 1 min) × 32 cycles; 72 °C for 10 min
	A2R	5′TCACACGGGTAGGCGCCGTTGATCCTCGTG 3′		
*pvdhps*	2 B F	5′AATGGCAAGTGATGGGGCGAGCGTGATT 3′	610	95 °C for 5 min; (94 °C for 40 s; 54 °C for 55 s; 72 °C for 1 min) × 35 cycles; 72 °C for 10 min
	2B R	5′CAGTCTGCACTCCCCGATGGCCGCGCCAC 3′		

### DNA sequencing of *pvdhfr* and *pvdhps* genes

After adequate PCR amplification of different amplicon, sequencing reactions were carried out in 3730xl genetic analyzer (Applied Biosystems) ≥2× coverage using an ABI Prism Big Dye Terminator cycle sequencing ready reaction kit. In the sequencing PCR reaction the final master mix volume was 20 µl, consisting of 1 µl of Terminator ready reaction mix (TRR), 3.2 pmol of gene-specific primer, and 0.5× sequencing buffer [28]. Sequencing experiments were carried out at the Indian Institute of Technology, Kharagpur (IIT, Kharagpur), and Sci Genome Company (Kochin). Electrophoregrams were visualized and analysed with CEQ 2000 genetic analysis system software (Beckman Coulter), and the sequencing traits were translated in the translation tool, available online at the Expert Protein Analysis System proteomic server [28]. Translated sequences were aligned online by the multiple sequence alignment tool ClustalW2 [28] and compared with the wild-type allele sequences (GenBank accession numbers, X98123 for *pvdhfr*, AY186730 for *pvdhps*). Polymorphisms of these two genes were confirmed by reading both the forward and reverse strands.

### Statistical analysis

The data were expressed as median ± SD values and % (percentage) variation; 95 % confidence intervals were calculated using Clopper-Pearson exact method. Graphical presentation of *dhfr* and *dhps* gene was carried out in Statistical Package, Origin 6.1.

## Results

### Polymorphism of *pvdhfr* gene

**Fig. 2:**
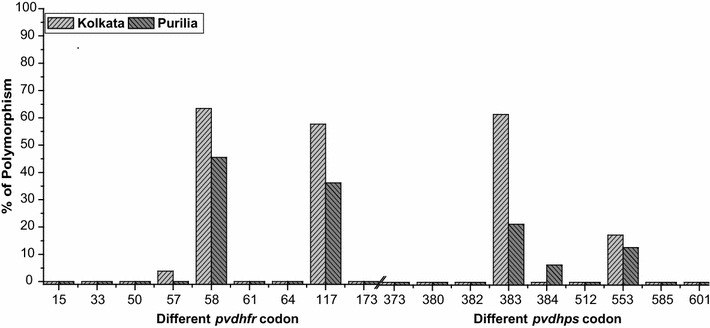
Frequency (% percentage) of different *pvdhfr* and *pvdhps* genotype

Monoclonal *P. vivax* infection was confirmed after allelic family-specific nested-PCR. A total of 99 *P. vivax*-positive patients confirmed by microscopy and PCR were recruited into the study population. The entire DHFR domain of *pvdhfr* gene was successfully sequenced in all 99 clinical *P. vivax* isolates. Predominance of wild type *dhfr* allele (n = 23, 48.94 %) was observed in Purulia whereas only 19 (36.54 %) isolates contained wild *dhfr* allele in KMC. Among these isolates, mutations were absent in codons 15, 33, 50, 61, 64, and 173. The frequency of non-synonymous polymorphism at S58R and S117N was higher among the isolates. In KMC 63.46 % of isolates presented mutant S58R codon, followed by mutant S117N codon (57.69 %) and F57L codon (3.84 %). Like KMC, polymorphism at codon S58R (42.55 %) was most prevalent in Purulia, followed by S117N polymorphism (36.17 %) (Fig. 2). Beside these known non-synonymous polymorphisms, three synonymous mutations at G38G {GGT → GGG; in five isolates (7.69 %)}, Y69Y {TAT → TAC; in three isolates (5.77 %)} and V134 V {GTC → GTT, in one isolate (1.92 %)} codon were identified in KMC. On the contrary in Purulia, a single synonymous mutation at E119E codon {GAG → GAA, in three isolates (6.38 %)} was observed.

### Mutation assessment of *pvdhps* gene

Different variant of *pvdhps* gene flanking was successfully amplified and sequenced in all 52 patients from KMC and 47 patients from Purulia. Three different non-synonymous and two synonymous mutations were found in the study site. Non-synonymous mutation at codon A383G was most prevalent (61.54 %) in KMC, followed by A553G mutation (17.30 %). *pfdhps* A383G polymorphism was far lower in Purulia. Only 21.27 % of isolates presented the mutant A383G allele followed by A553G mutant allele (12.77 %). Another non- synonymous mutation at P384L codon (6.38 %) was observed in Purulia (Fig. 2). No mutation was observed at codons S373T, E380K, S382A, K512E, V585G, and M601I. Beside these non-synonymous polymorphisms, two synonymous mutations were identified at R487R codon {CGC → CGG; in two isolates (3.85 %)}, and L600L {CTA → CTG; in three isolates (6.38 %)} codon in KMC. No synonymous mutation was identified in Purulia.

### Regional distribution of *pvdhfr* and *pvdhps* haplotype

**Fig. 3:**
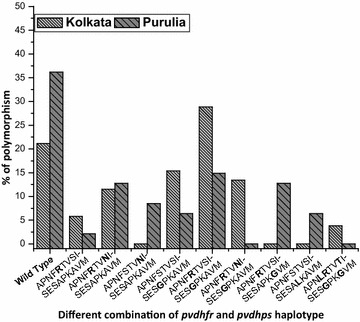
Frequency (% percentage) of different *pvdhfr*–*pvdhps* haplotype sequences in KMC and Purulia

Four different *pvdhfr* haplotypes were identified in KMC as well as in Purulia. In KMC, double mutant *dhfr* A_15_P_33_N_50_F_57_**R**_**58**_T_61_V_64_**N**_**117**_I_173_ allele (53.85 %) was frequently found after wild type A_15_P_33_N_50_F_57_S_58_T_61_V_64_S_117_I_173_ haplotype (36.54 %). In Purulia, wild type A_15_P_33_N_50_F_57_S_58_T_61_V_64_S_117_I_173_ allele was commonly (48.94 %) observed *pvdhfr* haplotype, followed by double mutant A_15_P_33_N_50_F_57_**R**_**58**_T_61_V_64_**N**_**117**_I_17_ allele (27.66 %). In KMC only 5.77 % of isolates represented *pvdhfr* single mutant A_15_P_33_N_50_F_57_**R**_**58**_T_61_V_64_ S_117_I_173_ allele whereas 14.89 % isolates in Purulia contained this single mutant allele. Interestingly, 8.51 % of isolates presented the single mutant S117 N codon, i.e., A_15_P_33_N_50_F_57_S_58_T_61_V_64_**N**_**117**_I_173_ allele in Purulia. In Purulia, 23.40 % of isolates contained single *pvdhfr* mutation which was far higher than KMC (Table 3). In KMC, triple mutant A_15_P_33_N_50_**L**_**57**_**R**_**58**_T_61_V_64_**N**_**117**_I_173_ allele was found in 3.84 % of isolates. No isolate was found to contain quadruple or quintuple *pvdhfr* mutations (Fig. 3).

In the case of *pvdhps* gene, three different haplotypes (wild type, 383G, 383G + 553G) were identified in KMC and four different haplotypes (wild type, 383G, 553G, 384L) in Purulia. Single mutant *pvdhps* S_373_E_380_S_382_**G**_**383**_P_384_K_512_A_553_V_585_M_601_ haplotype (44.23 %) was most predominant haplotype in KMC, followed by wild type *pvdhps* S_373_E_380_S_382_A_383_P_384_K_512_A_553_V_585_M_601_ allele (38.46 %). To the contrary, in Purulia, wild type *pvdhps* S_373_E_380_S_382_A_383_P_384_K_512_A_553_V_585_M_601_ allele (59.57 %) was highly prevalent *pvdhps* haplotype after single mutant *pvdhps* S_373_E_380_S_382_**G**_**383**_P_384_K_512_A_553_V_585_M_601_ allele (21.27 %). Interestingly, 17.31 % of isolates in KMC had represented the double mutant S_373_E_380_S_382_**G**_**383**_P_384_K_512_**G**_**553**_V_585_M_601_ allele but it was absent in isolates collected from Purulia (Fig. 3; Table 3).

### Variation and distribution of different tandem repeat

Two different tandem repeat variants were found, depending on deletion/insertion of six amino acids (GDNTSG) at *pvdhfr* gene. The type 2 tandem repeat of *pvdhfr* gene was the most common repeat polymorph observed in KMC (80.77 %) as well as in Purulia (100 %). Monomorphic tandem repeat variant was observed in Purulia, whereas polymorphic tandem repeats were found in isolates of KMC.

In *pvdhps* gene, three different tandem repeats variants were identified on the basis of deletion/insertion of seven amino acid repeat (GEAKLTN). Type B tandem repeat of *pvdhps* gene was prevalent in the study site in KMC (89.36 %) as well as in Purulia (63.47 %). Two tandem repeats were identified in isolates from KMC and three in Purulia. Distribution of tandem repeat variants in the study sites are presented in Table 2.

**Table 2:** Region wise distribution of tandem repeat variants of DHFR and DHPS gene

Gene	Type	Tandem repeat	KMC (%)	Purulia (%)
Dhfr	Type 1	GDNTSGGDNTHGGDNTHGGDNADKL	19.23	0.00
	Type 2	GDNTSGGDNTHG ------------GDNADKL	80.77	100
Dhps	Type A	GEAKLTNGEGKLTNGEAKLTNGEGKLTNG EAKLTNGEGKLTNGDAKLTNGDSKLTNG	10.64	21.15
	Type B	GEAKLTNGEGKLTNGEAKLTNGEGKLTNG -------------------------------------DSKLTNG	89.36	63.47
	Type C	GEAKLTNGEGKLTNGEAKLTNGEGKLTNG EAKLTNGEGKLTN -------------GDSKLTNG	0.00	15.38

### Combination mutation of *pvdhfr* and *pvdhps* gene and emergence of quadruple and quintuple mutant allele

A total of ten different *pvdhfr*–*pvdhps* two-locus haplotype combinations were identified among the total 99 isolates (Fig. 3). Seven different *pvdhfr*–*pvdhps* haplotype combinations (G1, G2, G3, G5, G6, G7, G10) were found in KMC and eight different haplotype combinations (G1, G2, G3, G4, G5, G6, G8, G9) were observed in Purulia (Table 3). Eleven isolates (21.15 %) in KMC and 17 isolates (36.17 %) in Purulia respectively presented the wild type two-locus genotypes, i.e., G1 genotype. A large number of isolates (28.84 % of isolates in KMC and 14.89 % of isolates in Purulia) had represented the double mutant *pvdhfr* and single mutant *pvdhps* (G6) allele. Haplotype data revealed that *pvdhfr*–*pvdhps* combination mutation begun to increase in number in KMC compared to Purulia. Some 13.46 % of isolates in KMC contained double mutation in both *pvdhfr* and *pvdhps* (G7) gene (quadruple mutation in two-locus combinations) but this haplotype was not observed in Purulia. Interestingly, in KMC, a very low proportion of quintuple mutation in two-locus combination (G10 genotype, triple mutation in *dhfr* gene and double mutation in *dhps* gene) was observed in 3.84 % (n = 2) of isolates but this haplotype was absent in isolates of Purulia (Table 3).

**Table 3:** Distribution of different *pvdhfr* and *pvdhps* combination haplotype Kolkata and Purulia

Genotype	Isolates in KMC	Isolates in P	PVDHFR									PVDHPS									Allele type
			15	33	50	57	58	61	64	117	173	373	380	382	383	384	512	553	585	601	
G1	11	17	A	P	N	F	S	T	V	S	I	S	E	S	A	P	K	A	V	M	Wild
G2	3	1	A	P	N	F	**R**	T	V	S	I	S	E	S	A	P	K	A	V	M	SM
G3	6	6	A	P	N	F	**R**	T	V	**N**	I	S	E	S	A	P	K	A	V	M	DM
G4	0	4	A	P	N	F	S	T	V	**N**	I	S	E	S	A	P	K	A	V	M	SM
G5	8	3	A	P	N	F	S	T	V	S	I	S	E	S	**G**	P	K	A	V	M	SM
G6	15	7	A	P	N	F	**R**	T	V	**N**	I	S	E	S	**G**	P	K	A	V	M	TM
G7	7	0	A	P	N	F	**R**	T	V	**N**	I	S	E	S	**G**	P	K	**G**	V	M	QM
G8	0	6	A	P	N	F	**R**	T	V	S	I	S	E	S	A	P	K	**G**	V	M	DM
G9	0	3	A	P	N	F	S	T	V	S	I	S	E	S	A	**L**	K	A	V	M	SM
G10	2	0	A	P	N	**L**	**R**	T	V	**N**	I	S	E	S	**G**	P	K	**G**	V	M	QTM

Bold amino acids are the mutant codon

SM, DM, TM, QM and QTM are respectively denote as single mutant, double mutant, triple mutant, quadruple mutant and quintuple mutant allele

## Discussion

Genetic diversity in *Plasmodium* is well known in India as both *P. falciparum* and *P. vivax* are prevalent in the same ecological niche. As *P. falciparum* and *P. vivax* parasites co-exist in this region and both have the same drug target against antifolate drug [7–11], the risk of resistance development is high. Polymorphisms in different variants of *pvdhfr* and *pvdhps* genes were known to be associated with antifolate resistance [8, 9]. However, mathematical modelling, in vitro experiments, and transcriptomic studies all suggest that reduced susceptibility to SP is solely related to *pvdhfr* and *pvdhps* [8, 13, 15, 19]. Therefore, genotyping of these candidate gene markers of *P. vivax* may elucidate trends of SP resistance in India.

The predominance of *pvdhfr* double mutant 58R + 117N (53.84 % in KMC and 27.65 % in Purulia) polymorphism was observed followed by single mutant S58R and S117N mutation. Non-synonymous 57L mutation was detected in combination with 58R + 117N in low frequency (3.85 %). These findings were similar to those reported from different parts of India [7, 20], Pakistan [29, 30], Afghanistan [31], China [32], Nepal [33], Thailand [18, 34, 35], and Indonesia [36]. In the study did not identify any polymorphism at codons A15, P33L, N50I, T61M, V64L, and I173L of *pvdhfr* gene, as previously reported from different parts of India [20, 22, 37]. It was postulated that *pvdhfr* S117N mutation might occur first, followed by the S58R mutation. Polymorphisms at codons F57L and T61M might take place independently with an increase in drug pressure [14]. The triple (L_57_R_58_N_117_) and quadruple (L_57_R_58_M_61_N_117_) *pvdhfr* polymorphism possessed progressive tolerance in *P. vivax* to SP, thus these genotypes were associated with high risk of therapeutic SP failure [35]. Unlike previous reports in India [20, 21], no quadruple mutation was observed in study site, although only two isolates (3.85 %) in KMC had represented the triple mutant allele, which was previously not observed in KMC [7].

Different studies from various geographical locations imply that polymorphism of *dhfr* gene was associated with tandem repeat polymorphism. In the study, it was observed that predominance of double *dhfr* mutation was associated with type-2 tandem repeat variant, which strongly supported the previous report in India [21]. On the contrary, previous reports from different geographical regions suggested that type-1 tandem repeat variant was highly associated with triple or quadruple mutant *dhfr* alleles [17, 21]. Similarly, not a single type-1 tandem repeat was identified in Purulia where no triple or quadruple mutations were observed, although a single exception to this was previously observed in Myanmar [38]. In the case of *dhps* gene, type B tandem repeat was prevalent in KMC as well as in Purulia, which was previously reported in different parts of India and Pakistan [29, 39]. Previous genome-wide analysis suggested that the type B tandem repeat was generally associated with wild type *pvdhps* gene, as happened in the study site, whereas the existence of double mutant *pvdhps* (383G + 553G) haplotype was generally associated with type A tandem repeat [29, 39]. Thus, the tandem repeat could be used as a molecular marker to predict the risk mutant genotypes that confer higher level resistance.

Polymorphism in *dhps* gene was higher in KMC than in Purulia isolates but there were some similarities in both places. In the case of *pvdhps* gene, polymorphisms were mainly observed at codons A383G and A553G. Unlike a previous report from KMC [7], double *dhps* mutation at 383G + 553G was predominantly observed in KMC. Absence of double *dhps* mutation (A383G + A553G) in Purulia indicated about there was less drug pressure of SP, in contrast to KMC. Predominance of double *dhps* 383G + 553G mutations were previously observed in isolates from Asaam, Nadiad and Tamil Nadu [20, 21]. It now proved that polymorphism at A383G and A553G were solely responsible for sulfadoxine resistance in vitro as well as in vivo as they possessed the sulfadoxine binding site in *dhps* gene, additional mutations at E380 K, S382A, P384L, K512E, V585G, and M601I codons confer higher levels of sulfadoxine resistance [18, 19].

In case of combination mutation of *pvdhfr* and *pvdhps* gene, it was clearly observed that the proportion of triple mutation (R_58_N_117_–G_383_), quadruple mutation (R_58_N_117_–G_383_G_553_), and quintuple mutation (L_57_R_58_N_117_–G_383_G_553_) were higher in KMC than Purulia. The probability for these major variations might be embedded in the basis of geographical variations of parasite infection. It was reported that *P. falciparum* + *P. vivax* mixed infection was highest in KMC whereas not a single case of *P. falciparum* + *P. vivax* mixed infection was observed in Purulia [22]. Thus, the parasite from KMC might possess higher SP partial pressure than those isolates from Purulia. Secondly, KMC was the gateway to Southeast Asia in India and the mixed population was very high in that part of India, which might produce westward movement of CQ and SP-resistant parasites from Greater Mekong Sub-region to Africa through India [40, 41].

In India, SP was never prescribed against *P. vivax* [7]; the emergence of point mutations in *pvdhfr* and *pvdhps* genes was quite unexpected. Exposure of *P. vivax* to SP might arise due to several reasons: first, *P. falciparum* and *P. vivax* mixed infections were very common here; artesunate plus SP was generally recommended in those patients and thus, *P. vivax* parasites may be directly exposed to SP drug pressure. In the study site, the proportion of *P. falciparum* isolates with increased *pfdhfr* and *pfdhps* polymorphisms has been identified over the past decade [10–12]. The increase in the number of *P. falciparum* + *P. vivax* co-infection might exert some selection pressure of SP over the *P. vivax* population; secondly, there could be a situation where a clinician recommended an anti-malarial drug depending upon the clinical grounds but with a lack of immediate diagnosis, as well as erroneous diagnosis of the parasite species; thirdly, presumptive treatment of malaria without proper diagnosis and use of SP combination by private practitioners could not be ruled out [21, 34]. Emergence and subsequent spread of multidrug-resistant parasites is a problem for malaria control and elimination programme. It is essential to identify and assess drug resistance markers in a regular, synchronous manner to recognize drug-resistant areas on the basis of candidate gene polymorphism analysis, which ultimately helps to manage suitable anti-malarial drug policy.

## Conclusion

The findings suggest that *P. vivax* parasites in rural Purulia may still be susceptible to SP, but additional caution should be taken for treatment of vivax malaria in KMC to limit blooming of quadruple and quintuple mutant allele in the remainder of West Bengal, India. Synchronized research, surveillance and containment strategies are essential to optimize the current use of anti-malarial drugs to limit resistance and to understand the proper genetic lineage of resistant parasites.

## Abbreviations

API: annual parasite index
CQ: chloroquine
DHFR: dihydrofolate reductase
DHPS: dihydropteroate synthase
DNA: deoxyribonucleic acid
dNTPs: deoxynucleotides
DNase: deoxyribonuclase
EDTA: ethylene diamine tetra acetic acid
IC: inhibitory concentration
KCl: potassium chloride
KMC: Kolkata Municipal Corporation
mg: milligram
MgCl_2_: Magnesium Chloride
ml: millilitre
mM: millimolar
NVBDCP: National Vector Borne Disease Control Programme
ng: nanogram
OD: optical density
PCR: polymerase chain reaction
Pf: *Plasmodium falciparum*
Pv: *Plasmodium vivax*
RBC: red blood cells
RDT: rapid diagnostic test
rpm: rounds per minute
RPMI 1640: Roswell Park Memorial Institute medium 1640
SEM: standard error of mean
SNPs: single nuclear polymorphisms
SP: sulfadoxine-pyrimethamine
TBE: tris-borate EDTA
TE: tris-EDTA
TRR: terminator ready reaction
WHO: World Health Organization
μg: microgram
μl: microliter
μM: micromolar
